# Mortality Rate for Children under 5 Years of Age in Zhejiang Province, China from 1997 to 2012

**DOI:** 10.1371/journal.pone.0127770

**Published:** 2015-06-04

**Authors:** Weifang Zhang, Dingwan Chen, Yanhua Xu, Rulai Yang, Zhengyan Zhao

**Affiliations:** 1 Department of Administration, Children’s Hospital, Zhejiang University School of Medicine, Hangzhou, 310003, China; 2 Department of Public Health, Zhejiang Medical College, Hangzhou, 310003, China; FIOCRUZ, BRAZIL

## Abstract

**Objectives:**

This is a population based descriptive study that examined the trends in childhood mortality among under five children and the major causes under five mortality in Zhejiang Province, China.

**Methods:**

A population-based survey was conducted through a province-level surveillance network. The mortality rate and leading causes of death for children under 5 years of age were analyzed. The trend in the mortality rate for children under five and cause-specific mortality rates were analyzed by chi-square with SPSS 13.0 software.

**Results:**

In Zhejiang Province, during 1997-2012, mortality rates in neonates, postneonatal infants, and children under 5 years were reduced by 64.2% (from 7.85 to 2.81 per 1000 livebirths), 66.7% (from 12.73 to 4.24 per 1000 livebirths), and 63% (from 15.76 to 5.85 per 1000 livebirths), respectively. The mortality rates in children under 5 years of age decreased by 59.5% (from 11.09 to 4.49 per 1000 livebirths) and 65.8% (from 19.30 to 6.61 per 1000 livebirths) in urban and rural areas, respectively. Prematurity/low birth weight and congenital heart disease were in the top five causes of death in children under 5 years of age during 1997-2012.

**Conclusions:**

Zhejiang province has achieved great progress in the reduction of mortality rates in children under five-years-old during the past two decades. The future tasks on reduction of mortality rate still rely on how to improve the management of premature birth/low birth weight, reduce birth defects and prevent accidental deaths in Zhejiang Province.

## Introduction

China has made progress in reducing the number of child deaths during the past decades, and has achieved the Millennium Development Goal 4 (MDG4) in the Process[1–4]. Millennium Development Goal 4 calls for an annual rate of reduction of the under-five mortality rate of 4.4% between 1990 and 2015. The nationwide mortality rate for children under 5 years of age in China decreased from 39.7 per 1000 livebirths in 2000 to 20.6 per livebirths in 2006[5] and 16.4 in 2010 [6]. China still has not attained a desired level of child health compared with the wealthy industrialised countries where the U5MR was 6‰ in 2007 [7].

Since the mid-1980s, China has begun to establish the Children Death Report System and Surveillance system. The Children Death Report System covered 31 provinces, autonomous regions and municipalities with 337 national-level death surveillance regions for children under-five years. In 2000, the Chinese government financed and implemented the Reducing Maternal Mortality and Eliminating Neonatal Tetanus Project in 378 rural counties in West China. By 2004, this project had expanded to 1000 rural counties in Mid-West China[4].The Project has played a significant role in reducing the infant mortality rate and improving the health of children living in rural areas.

Zhejiang Province, one of the most commercial and richest provinces in China, includes 90 counties in 11 administrative regions (Hangzhou, Ningbo, Wenzhou, Jiaxing, Huzhou, Shaoxing, Jinhua, Quzhou, Zhoushan, Taizhou, and Lishui). The monitoring of maternal health care services has been performed since the early 1990s. The local governments of Zhejiang Province formulated related regulations to ensure the standard reporting system, therefore improving the mortality surveillance procedure. The mortality rate in Zhejiang Province has been decreased in the past decades, however still higher than some more developed cities and regions like Beijing city.[8] Identification of the main causes of childhood mortality will be beneficial to further preventing deaths among young children and reduce the mortality rate. Therefore, this epidemiologic study aimed to analyze the mortality rate and main death causes for children under 5 years of age in Zhejiang Province from 1997 to 2012 and provide some points for reducing mortality rate.

## Methods

### Surveillance of the mortality rate in children

This epidemiologic study was approved by Ethical Committee, Children’s Hospital, Zhejiang University School of Medicine (IRB no. 2013118.). All patient records/information was anonymized and de-identified prior to analysis. The surveillance program of the mortality rates in children was initiated in Zhejiang Province in the 1990s. During the past two decades, a three-level maternal and child health care network (province level-city/district level-county level) has been developed and an integrated child mortality surveillance system has been established with a coverage of 30 out of 90 counties of Zhejiang Province. The covered surveillance sites were selected randomly. In 2005, about 25% of the people living in the 30 surveyed counties was monitored; while all persons living in the surveillance sites (nearly a main population of 8,243,000) have been monitored since 2006. The surveillance areas were differentiated as urban and rural areas. During 1997–2012, totally 1.47 million livebirths and 12,413 deaths were under surveillance. A livebirth was defined as a fetus born after 28 gestational weeks with at least one of the following vital signs: heartbeat, breathing, pulsation of the umbilical cord, or contraction of voluntary muscle. Categorization of causes of death was in accordance with the International Categorization of Diseases (ICD-9). Disease names were based on the book ‘Practical Pediatrics’[9].

Data collection was done by well-trained staffs of local maternal and children health care facilities which constitutes the three-level maternal and children’s health care network. These data were then reported to higher-level surveillance specialists. The flow of data collection of community-district-city for urban areas or village-township-county rural areas was followed. All information was reported and gathered to the Province Surveillance Office, Children’s Hospital, Zhejiang University School of Medicine.

### Quality control

We implemented the Provincial Maternal and Children’s Health Surveillance Quality Control Specialists Group. Quality control has been conducted in all surveillance sites across the Province at regular times since 1997. The county-level maternal and health care facilities were responsible for data collecting, checking, and follow-up. Quality control was done at 50% countyship-level surveillance sites every six months. Spot checks at surveillance areas (25%) were randomly carried out by the maternal and children healthcare institution at the city and province level every year. Quality control focused on life indicators and information organization. The life indicators included livebirths, neonatal deaths, infant deaths, and under-five deaths. Information organization included staff, information gathering staff, training, healthcare information network establishment and related knowledge. All data will be corrected by the control results, which making the data valid.

### Statistical analysis

The mortality rate was calculated by the number of deaths among children under five per 1,000 livebirths. The cause-specific mortality rate was calculated by the number of deaths among children under 5 years of age caused by a specific disease per 100,000 livebirths. Annual average rate of reduction of mortality in children under five years was analyzed between 1997 and 2006, 2006 and 2012. The trend in the mortality rate for children under five and cause-specific mortality rates were analyzed by chi-square with SPSS 13.0 software. A value of *p*<0.05 is considered significant.

## Results

### Overall mortality rates in children under five years

**Fig 1:**
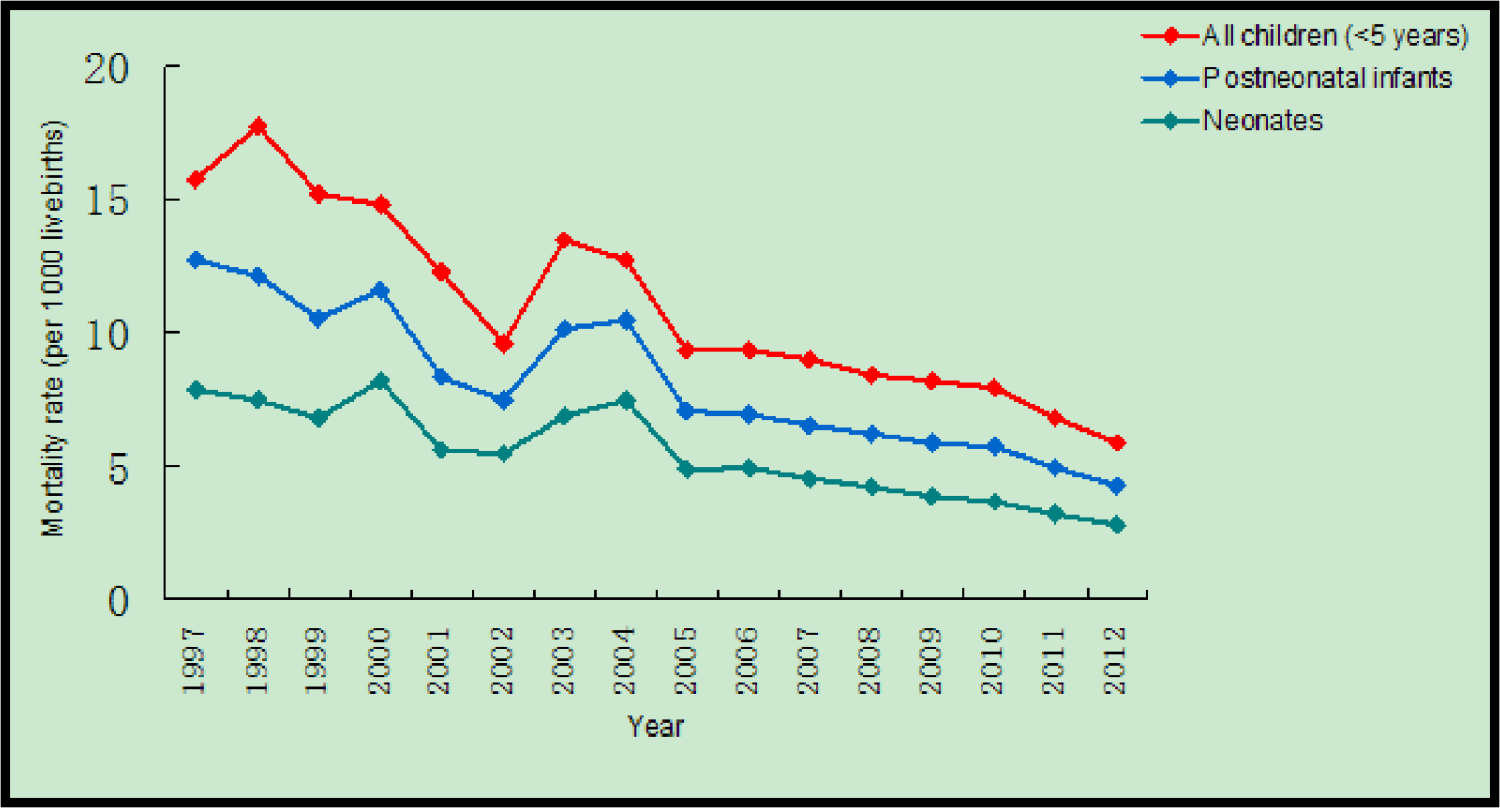
Trends of mortality rates in Zhejiang Province, China (1997–2012) in neonates, postneonatal infants and all children (< 5 years).

**Fig 2:**
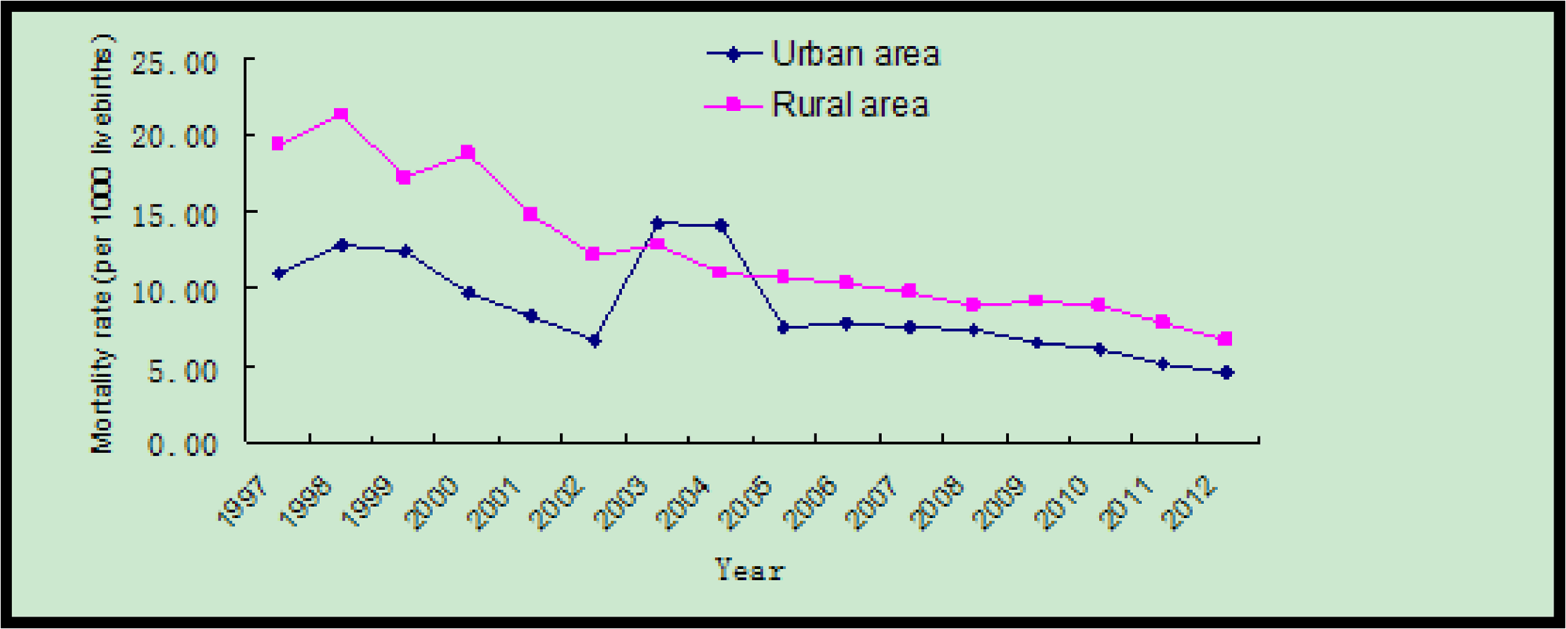
Difference of mortality rates of children under five years in urban and rural areas in Zhejiang Province, China (1997–2012).

In Zhejiang Province, during 1997–2012, mortality rates in neonates, postneonatal infants, and children under five years were reduced by 64.2% (from 7.8 to 2.8 per 1000 livebirths), 66.7% (from 12.7 to 4.2 per 1000 livebirths), and 63% (from 15.7 to 5.8 per 1000 livebirths), respectively (Fig 1). The mortality rates in children under 5 years of age decreased by 59.5% (from 11.0 to 4.4 per 1000 livebirths) and 65.8% (from 19.3 to 6.6 per 1000 livebirths) in urban and rural areas, respectively. The reduction of mortality rate in children <5 years was significant during 1997–2006 (annual average rate of reduction, AAPR: 5.1% [95%CI: 4.7%-5.5%]) and 2006–2012 (AAPR: 8.1%, 95% CI: 7.4%-8.8%) (Table 1). The mortality rate for children under 5 years was higher in rural areas than urban areas during the study period except in the years of 2004 and 2005. The largest difference in the mortality rate was in 2000 with a 1.93 times higher mortality rate in rural areas than urban areas (Fig 2).

**Table 1:** Comparison of mortality rates in children aged under 5 years (U5MR) in Zhejiang Province, China in 1997, 2006, and 2012 (per 1000 livebirths).

	1997U5MR (95% CI)	2006U5MR (95% CI)	AARR (%)	P value	2006U5MR (95% CI)	2012U5MR (95% CI)	AARR (%)	P value
Province wide	15.8 (13.7–17.9)	9.3 (7.2–11.4)	-5.1 (-5.5–-4.7)	<0.001	9.3 (7.2–11.4)	5.9 (5.3–6.5)	-8.1 (-8.8–-7.4)	<0.001
Urban areas	11.1 (8.4–13.8)	7.7 (5.0–10.4)	-3.7 (-4.0–-3.4)	<0.001	7.7 (5.0–10.4)	4.5 (3.5–5.5)	-9.5 (-9.9–-9.1)	<0.001
Rural areas	19.3 (16.3–22.3)	10.2 (7.2–13.2)	-6.1 (-6.5–5.7)	<0.001	10.3 (7.2–13.2)	6.6 (5.7–7.5)	-7.7 (-7.9–-7.5)	<0.001

AARR: annual average rate of reduction; CI: confidence interval.

### Cause-specific mortality rates and leading causes of death in children under 5 years of age

**Fig 3:**
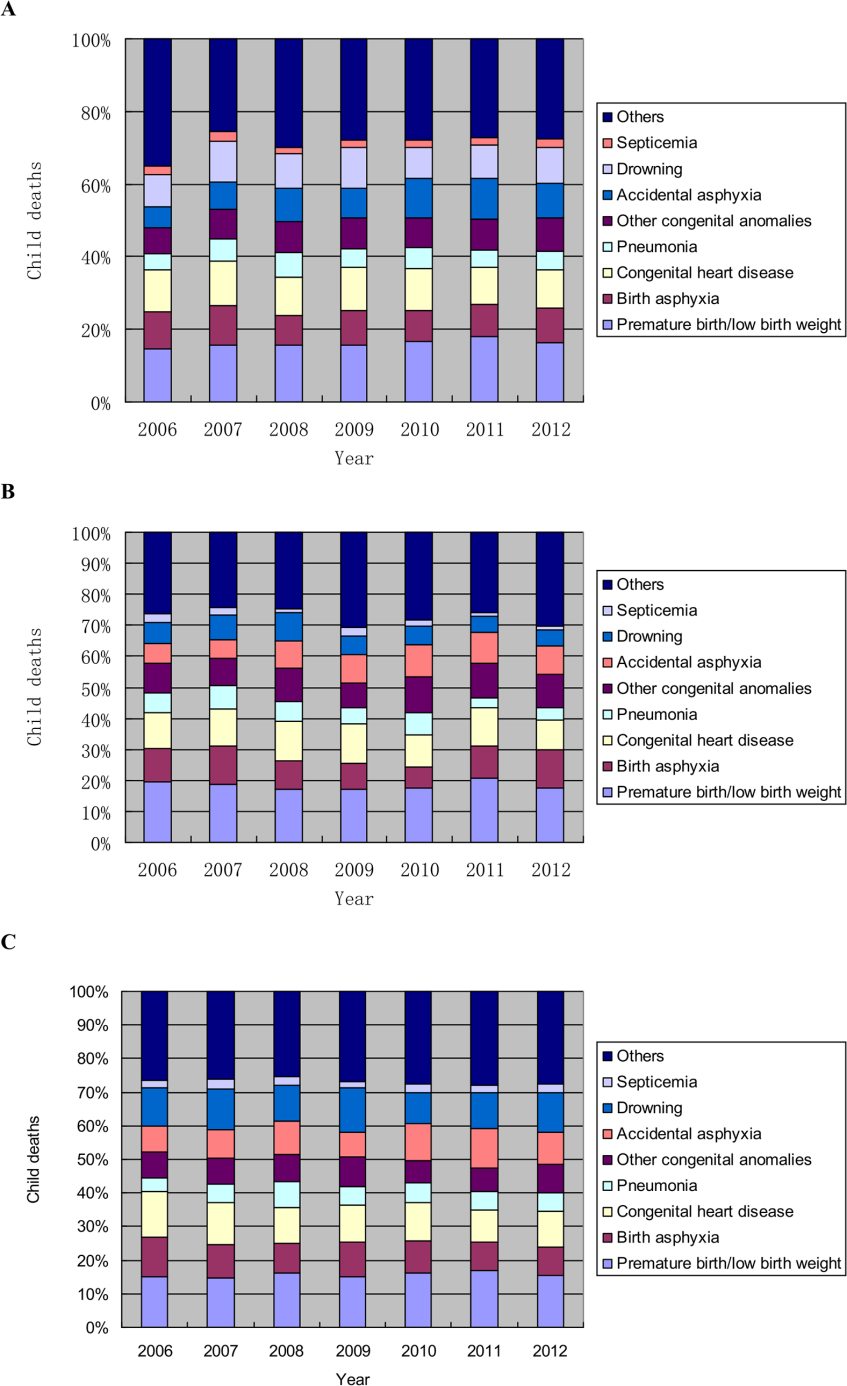
Trends in proportional contribution of most common causes of child deaths in Zhejiang Province during 2006–2012. A: Province-level; B: Urban area; C: Rural area.

Tables 2–4 shows the changes of cause-specific mortality rates and leading causes of death in children under 5 years of age from 1997 to 2005. Fig 3 shows the the changes of cause-specific mortality rates and leading causes of death in children under 5 years of age from 2006 to 2012. Prematurity/low birth weight and congenital heart disease were in the top five causes of death in children under 5 years of age during 1997–2012. Deaths due to prematurity/low birth weight and congenital heart disease were reduced by 46% and 71.1% respectively, from 1997 to 2012. During 2002–2012, prematurity/low birth weight was the leading cause of death in children under 5 years of age. During the period of 16 years, mortality rate due to birth asphyxia, congenital heart disease, pneumonia and other congenital anomalies reduced dramatically; mortality rate due to accidental asphyxia and drowning has no dramatic improvement. Accidental asphyxia has been in the top five causes of mortality for three consecutive years from 2010 to 2012, and drowning was still in the top five leading causes of mortality.

**Table 2:** The leading causes of under-five mortality at province level in Zhejiang Province, China (1997–2005).

Causes of death	Cause-specific mortality rate (per 100,000 live births) (number of deaths)								
	1997 (15,165)	1998 (12,961)	1999 (12,984)	2000(13,691)	2001(12,821)	2002(26,944)	2003(29,200)	2004(45,431)	2005(38,478)
Premature birth/low birth weight	178.0 (27)	223.7 (29)	200.2 (26)	189.9 (26)	155.9 (20)	154.4 (42)	202.0 (59)	193.7 (88)	137.7 (53)
Birth asphyxia	164.9 (25)	154.3 (20)	169.4 (22)	197.2 (27)	101.4 (13)	-	130.1(38)	134.2 (61)	98.7 (38)
Congenital heart disease	211.0 (31)	223.7 (29)	146.3(19)	167.9(23)	202.7 (26)	135.6 (37)	191.7 (56)	167.2 (76)	90.9(35)
Pneumonia	125.3 (19)	223.7 (29)	-	131.4 (18)	-	60.2 (16)	-	96.8 (44)	-
Other congenital anomalies	171.4 (26)	185.2 (24)	130.9(17)	204.5(28)	163.7 (21)	94.18(25)	102.7 (30)	-	96.1 (37)
Accidental asphyxia	-	-	-	-	-	-	-	107.8 (49)	77.9 (30)
Drowning	-	-	207.9 (27)	-	140.3 (18)	90.4 (24)	126.7 (37)	-	77.9(30)

**Table 3:** The leading causes of under-five mortality at urban areas in Zhejiang Province, China (1997–2005).

Causes of death	Cause-specific mortality (per 100,000 live births) (number of deaths)								
	1997 (6405)	1998 (5514)	1999 (5620)	2000 (6054)	2001 (4994)	2002 (12683)	2003 (12765)	2004 (25635)	2005 (17128)
Premature birth/low birth weight	140.5 (90)	253.9 (140)	284.7(160)	148.6 (90)	200.2 (100)	105.8 (134)	203.6 (260)	214.5 (549)	140.1 (240)
Birth asphyxia	62.5 (40)	72.5 (40)	71.2(40)	132.1 (80)	-	56.9 (72)	141.0 (180)	171.6 (440)	87.58 (250)
Congenital heart disease	203 (13)	-	195.7 (110)	181.7(110)	120.1 (60)	122.1 (154)	195.8 (250)	183.3 (470)	110.9(190)
Pneumonia	-	126.9 (70)	-	49.5 (30)	-	-	141.0 (180)	136.3 (470)	64.2(110)
Other congenital anomalies	171.7 (110)	235.8 (130)	89.0(50)	247.7 (150)	180.2 (90)	65.13 (82)	101.8 (130)	-	46.7(80)
Accidental asphyxia	78.1 (50)	-	-	-	40.05(20)	40.71 (51)	-	101.42(260)	46.7(80)
Drowning	-	54.4 (30)	53.4 (30)	-	60.07 (30)	-	94.01 (120)	-	-

**Table 4:** The leading causes of under-five mortality at rural areas in Zhejiang Province, China (1997–2005).

Causes of death	Cause-specific mortality (per 100,000 live births) (number of deaths)								
	1997 (8706)	1998 (7447)	1999 (7364)	2000 (7637)	2001 (7827)	2002 (14,261)	2003(16,435)	2004(19,796)	2005(21,350)
Premature birth/low birth weight	205.5 (179)	-	-	137.8 (105)	222.6 (174)	127.7 (182)	196.3 (322)	166.7 (330)	135.8 (290)
Birth asphyxia	239.7 (209)	214.9 (187)	201.4 (148)	244.4 (187)	248.7 (195)	-	119.2 (196)	121.6 (241)	107.7 (230)
Congenital heart disease	216.9(189)	-	201.4(148)	-	157.1 (123)	255.5 (364)	147.2 (242)	146.4 (290)	-
Pneumonia	194.1(169)	295.4 (257)	-	149.4 (114)	196.4 (154)	-	84.1 (138)	-	98.3 (210)
Other congenital anomalies	-	-	147.7 (109)	163.0(124)	170.2 (133)	153.3 (219)	103.4 (169)	96.0 (190)	135.8 (290)
Accidental asphyxia	-	-	107.4 (79)	-	-	114.9 (164)	84.1 (138)	116.1 (230)	103.0 (220)
Drowning	138.5 (121)	214.9 (187)	-	325.9 (249)	157.1 (123)	191.6 (273)	126.2 (207)	136.3 (270)	126.4(270)

In urban areas, deaths due to premature birth/low birth weight and congenital heart diseases from 1997 to 2012 decreased by 41.8% and 78.3%, respectively. However, deaths due to birth asphyxia were only reduced by 7.52%. In rural areas, the deaths due to premature birth/low birth weight and congenital heart diseases from 1997 to 2012 decreased by 49.8% and 72.7%, respectively. Deaths due to birth asphyxia were reduced by 67.6%. Drowning was a leading cause of death in rural areas during the study period except 1999 (Table 4).

At province level, the five leading causes of death in children under five years were congenital heart diseases, premature birth/low birth weight, other congenital anomalies, birth asphyxia and pneumonia in 1997; premature birth/low birth weight, congenital heart diseases, birth asphyxia, drowning, and other congenital anomalies in 2006; and premature birth/low birth weight, congenital heart diseases, drowning, accidental asphyxia, and birth asphyxia in 2012. In 2006, drowning became one of the five leading causes instead of pneumonia. Compared to 2006, accidental asphyxia was one of the leading causes instead of other congenital anomalies in 2012 (Table 2).

### Difference in leading causes of death in children under five years between urban and rural areas

Table 5 shows the difference in leading causes of death in children under five years between urban and rural areas. The cause specific death rates were higher in rural areas than in urban areas in all years. In urban areas, the leading five causes of death were congenital heart diseases, other congenital anomalies, premature birth/low birth weight, accidental asphyxia, and birth asphyxia in 1997; premature birth/low birth weight, congenital heart diseases, birth asphyxia, other congenital anomalies and drowning in 2006; and premature birth/low birth weight, birth asphyxia, other congenital anomalies, congenital heart diseases and accidental asphyxia in 2012. Pneumonia dropped out of the leading five. In rural areas, birth asphyxia, congenital heart diseases, premature birth/low birth weight, pneumonia, and drowning were the leading five causes of death in 1997; premature birth/low birth weight, congenital heart diseases, birth asphyxia, drowning and other congenital anomalies in 2006; and premature birth/low birth weight, drowning, congenital heart diseases, accidental asphyxia and other congenital anomalies in 2012. Birth asphyxia dropped out of the leading five and drowning became the second leading cause of the asphyxia in 2012.

**Table 5:** Comparison of top five leading causes of death in children under five years in Zhejiang Province, China in 1997, 2006, and 2012 (per 1000 live births).

	Top 1		Top 2		Top 3		Top 4		Top 5	
	Causes	Rates	Causes	Rates	Causes	Rates	Causes	Rates	Causes	Rates
**1997**										
Urban	CH	203	Other CA	171.7	Preterm and LBW	140.5	AA	78.1	BA	62.5
Rural	BA	239.7	CH	216.9	Preterm and LBW	205.5	Pneumonia	194.1	Drowning	138.5
**2006**										
Urban	Preterm and LBW	151.83	CH	89	BA	81.15	Other CA	73.3	Drowning	52.35
Rural	Preterm and LBW	156.88	CH	141.5	BA	121.51	Drowning	116.89	Other CA	79.98
**2012**										
Urban	Preterm and LBW	81.71	BA	57.82	Other CA	50.28	CH	44	AA	42.74
Rural	Preterm and LBW	103.11	Drowning	77.33	CH	70.36	AA	64.09	Other CA	55.04

CH: congenital heart disease; BA: birth asphyxia; CA: congenital anomalies; AA: accidental asphyxia; LBW: low birth weight.

## Discussion

From 1997 to 2012, the mortality rate of children under five years of age in Zhejiang Province, China had an overall decreasing trend; the mortality rate increased in 2003 and 2004, and it has been decreasing since 2005 and remaining at a low level through all the years. Since 2006, the entire population surveillance was implemented in Zhejiang Province; this change in methodology might remedy the limitation of volatile mortality rates which resulting from small samples, low coverage of livebirths and overestimation of mortality rate. In developed countries, public health surveillance systems are generally based on the entire population [10–14]. Incompletion of the registry system of public health causes data inaccuracy which is a serious problem in the surveillance.

From 1997–2012, the mortality rates of children under five years in Zhejiang Province were all much lower than that of whole country. The mortality rate of children under 5 years was 3.08, 2.76, and 5.85 times lower than that of the whole country in 2000, 2005, and 2012, respectively[15]. Among the 31 Provinces/direct-controlled municipalities/autonomous municipalities, the mortality rate of children under five years in Zhejiang Province is at a lower level; the mortality rate is slightly higher than that of Beijing city, but lower than most provinces and cities of China [15–20]. Our data also showed that neonatal mortality declined more than the under-five mortality, it may be due to specific interventions implemented for reducing neonatal mortality during the past two decades; the interventions include integrated community based maternal and neonatal care, improved antenatal health care and newborn resuscitation technique, exclusive breastfeeding, management of infections in newborns, and so on.

While the mortality rate of children under five years in Zhejiang Province decreased, the disparities between urban and rural populations were still very large. In 1997, 2006, and 2012, the mortality rates in rural areas were all significantly higher than those in the urban areas. The main reason may due to unequal health care access and facilities. The deaths occurring at home in rural areas was much higher than that in urban areas in Zhejiang Province. In 2006, per-person health expenditures for urban residents were much greater than those for rural residents[21,22]. Urban residents also had three times as many hospital beds and medical personnel per 1000 people [23]. The number of deaths which occurred at home in developed rural areas were lower than that of under-developed rural ones [23,24]. Further work should focus on how to reduce the disparity and improve the life quality of children from rural areas and immigrant populations.

In 2012, the leading five causes of deaths in children under five years of age were premature birth/low birth weight, congenital heart diseases, drowning, accidental asphyxia and birth asphyxia. However, premature birth/low birth weight, birth asphyxia, pneumonia, and accidental asphyxia were the leading five causes of deaths in children under five years countrywide. Premature birth/low birth weight has become the number one leading cause of death instead of birth asphyxia and congenital heart disease in children under five years in Zhejiang Province. A study in Beijing city revealed that premature birth/low birth weight may be due to advanced maternal age and multiple pregnancy by assisted reproduction techniques[24]; this study also suggested that multiple pregnancy will be more likely to cause preterm births and low birth weight infants.[24] For investigating the reasons of increased deaths due to premature birth/low birth weight, we consider to include maternity and pregnancy information in further data collection. Since 2009, China has implemented a new health care reform plan by including a wide range of basic medical insurance coverage for children and adolescents for the first time.[25] This health care reform has helped a lot on treating some major diseases such as congenital heart disease. Congenital heart disease was listed into the category of medical insurance since 2009. Deaths due to congenital heart disease have greatly decreased, but are still listed as the second leading cause. It is a great help that the government of Zhejiang province began reimbursing 90% of the medical expenses for children with leukemia and CHD since 2012.[26] We believe that deaths due to congenital heart disease will decrease more in the following years. In Zhejiang Province, birth defects are becoming the major cause of death in children under five years; we still need to strengthen prenatal diagnosis and perinatal health care particularly in the local maternity and children health care facilities. In contrast to country-wide with pneumonia as the second leading cause of death[25], pneumonia is no longer a leading cause of death in Zhejiang Province; however, deaths due to incidents such as accidental asphyxia are likely to to be increasing, which alerts us to enhance health care education (safe breeding and care for infants) and training (prevention and emergent management of accidental asphyxia) among parents or guardians. Drowning is a very important cause of death in children under five years, particularly in rural areas which is characteristic of Zhejiang Province as compared to other provinces [17–22]. In comparison with the urban areas, deaths due to accidents in rural areas were much higher. Drowning ranked as the second leading cause of death in children under five years in rural areas next to premature birth/low birth weight. This may be due to a scattered water-rich environment, lack of safety education, and the high numbers of leftover children without care of parents in rural areas. Accordingly, rural children should be considered as the key management population. A strategy to prevent accidental deaths has now become utmost important in reducing the mortality rate of children under five years in Zhejiang Province. Meanwhile, the government should also focus on premature birth/low birth weight which is the leading cause of death. The government should increase the health care allocation, reinforce health and safety education, and improve health interventions in rural areas.

There are limitations in this study. Firstly, due to the backward data storage system, part of the data before 2006 are missing. Therefore, some information can not be compared longitudinally. Secondly, not all the people of surveyed sites were included before 2006; therefore, low coverage of livebirths and overestimation of mortality rate existed; Third, the coverage of this data changed overtime which might have affected the observed trend in under-five mortaliy However, our study has strengths. Firstly, through this 16-year retrospective data analysis, we have revealed the trend of changes of mortality rate under five years and leading causes in Zhejiang Province. The results will be useful for the government to formulate corresponding regulations to reduce mortality rate under five years in the future. Zhejiang Province have implemented some effective measures and surveillance system to prevent deaths under five years; other areas or countries consider these measures to reduce mortality under five years further.

In conclusion, Zhejiang Province has achieved great progress in the reduction of mortality rates in children under five years of age during the past two decades. The progress may be related to social and economic development, maternal and children health-care system improvements, improved education of the population, increased personal and household wealth, insurance coverage and introduction of a series of interventions including vaccinations and newborn screenings. Decrease in the mortality rates still rely on how to improve the management of premature birth/low birth weight, reduction of the birth defects and prevention of accidental deaths in Zhejiang Province.
